# Three-Year Retained Endobronchial Push Pin in an Adolescent Causing Saccular Bronchiectasis: A Case Report and Review of the Literature

**DOI:** 10.3390/children13101395

**Published:** 2026-10-13

**Authors:** Stoyan S. Markov, Petya P. Markova, Kalina I. Madzarova-Nikolova

**Affiliations:** 1 Department of Otorhinolaryngology, Medical University of Plovdiv, 4002 Plovdiv, Bulgaria; kalina.madzarova@mu-plovdiv.bg; 2 Department of Otorhinolaryngology, “St. George” University Hospital Plovdiv, 4001 Plovdiv, Bulgaria; 3 Department of Pediatrics, Medical University of Plovdiv, 4002 Plovdiv, Bulgaria; petya.markova@mu-plovdiv.bg; 4 Department of Pediatrics, “St. George” University Hospital Plovdiv, 4001 Plovdiv, Bulgaria

**Keywords:** foreign body aspiration, bronchiectasis, bronchoscopy, adolescent, push pin, metal corrosion, delayed diagnosis, recurrent pneumonia

## Abstract

**Highlights:**

**What are the main findings?**

- A push pin retained for three years caused extensive local bronchial changes, including granulation tissue and multiple distal saccular bronchiectases.
- Prolonged retention was associated with marked corrosion and structural degradation of the metallic component, complicating bronchoscopic extraction.

**What are the implications of the main findings?**

- Chronic cough and recurrent or localized pulmonary infections in adolescents should prompt consideration of a retained endobronchial foreign body, even when the initial aspiration event was not recognized.
- Early CT assessment and diagnostic bronchoscopy may prevent irreversible bronchial and pulmonary damage caused by prolonged foreign body retention.

**Abstract:**

Background: Foreign body aspiration may remain undiagnosed for prolonged periods and lead to potentially irreversible pulmonary complications. The aim of this report was to present a rare case of a long-retained metallic endobronchial foreign body in an adolescent and to highlight the diagnostic and therapeutic challenges associated with delayed diagnosis. Case Presentation: A 16-year-old male was admitted with persistent cough and a three-year history of recurrent bronchitis and pneumonia treated mainly with antibiotics. Chest radiography revealed a radiopaque foreign body in the tracheobronchial tree. Computed tomography demonstrated a metallic, needle-shaped foreign body lodged in the right lower lobe bronchus, with multiple distal saccular bronchiectatic changes, some containing air–fluid levels. Following further questioning, the patient reported an episode of choking on a corkboard push pin three years earlier, which had not been disclosed at the time. Rigid bronchoscopy revealed the foreign body partially covered by granulation tissue. It was successfully removed using endoscopic forceps; however, part of the corroded metal tip fragmented during extraction. Purulent secretions distal to the obstruction were aspirated. The postoperative course was uncomplicated, although cough persisted and required further medical treatment. The patient did not attend follow-up visits and was lost to follow-up. Conclusions: Prolonged retention of an endobronchial foreign body may cause significant structural pulmonary damage, including bronchiectatic changes. Persistent cough and recurrent respiratory infections in children and adolescents should prompt consideration of an occult foreign body, even when the aspiration event is remote or initially unrecognized. Timely imaging and bronchoscopic evaluation may help limit the risk of potentially irreversible complications.

## 1. Introduction

Foreign body aspiration (FBA) remains a significant cause of morbidity and mortality worldwide and continues to represent an important clinical challenge despite substantial advances in diagnostic imaging and interventional bronchoscopy [1,2,3,4,5]. It is one of the most common pediatric emergencies requiring urgent bronchoscopic intervention, with approximately 80–90% of aspiration episodes occurring in children younger than three years of age because of immature swallowing coordination, incomplete dentition, and the tendency to explore objects orally [1,3,4]. In contrast, tracheobronchial foreign body aspiration in adolescents and adults is considerably less common and is usually associated with predisposing conditions such as neurological disorders, impaired protective airway reflexes, alcohol or drug intoxication, maxillofacial trauma, dental procedures, or accidental aspiration during eating [2,5,6].

The clinical manifestations of foreign body aspiration depend on the patient’s age, the anatomical location of the foreign body, its size, shape, composition, and the duration of endobronchial retention [1,2]. Acute aspiration is typically characterized by a sudden choking episode followed by persistent coughing, wheezing, dyspnea, stridor, or unilateral reduction in breath sounds. In many patients, particularly those with complete airway obstruction, the diagnosis is established promptly because of the dramatic onset of symptoms [1,3]. However, when the initial aspiration episode is mild, transient, or overlooked, patients may remain undiagnosed for prolonged periods, resulting in chronic respiratory symptoms that frequently mimic more common pulmonary diseases [2,5,7]. Consequently, many patients receive repeated courses of antibiotics, bronchodilators, or corticosteroids before the underlying diagnosis is finally established.

Although foreign body aspiration is generally regarded as an acute clinical event, delayed diagnosis remains a well-recognized problem, particularly in adolescents and adults. The absence of severe initial symptoms, failure to recognize or adequately investigate the aspiration episode, and the nonspecific nature of subsequent respiratory complaints frequently contribute to prolonged retention of the foreign body [2,5,6]. Even in patients who clearly recall the aspiration event, the diagnosis may be overlooked if the initial clinical examination and chest radiography are unremarkable or if symptoms temporarily improve following empirical medical treatment. Chronic cough, recurrent bronchitis, recurrent pneumonia, localized wheezing, hemoptysis, or treatment-resistant asthma are among the most frequently reported manifestations of retained endobronchial foreign bodies [2,5,6,8]. In some patients, the interval between aspiration and definitive diagnosis may extend from several months to many years, considerably increasing the risk of irreversible pulmonary injury [2,8].

Persistent bronchial obstruction initiates a cascade of pathological events including impaired mucociliary clearance, retention of secretions, recurrent bacterial infection, chronic inflammation, and progressive formation of granulation tissue surrounding the foreign body [7,9]. Repeated cycles of infection and inflammation progressively destroy the elastic and muscular components of the bronchial wall, eventually resulting in irreversible bronchial dilatation, the pathological hallmark of bronchiectasis [7,10]. The severity of pulmonary damage depends largely on the duration of foreign body retention, the degree of bronchial obstruction, the nature of the aspirated material, and the frequency of secondary infections. Previous studies have consistently demonstrated that prolonged diagnostic delay is associated with a significantly higher incidence of bronchiectasis, chronic atelectasis, post-obstructive pneumonia, lung abscess, bronchial stenosis, and irreversible destruction of the distal lung parenchyma, occasionally requiring pulmonary resection [7,9].

Radiological evaluation plays a central role in the diagnosis of retained tracheobronchial foreign bodies and in assessing the extent of secondary pulmonary damage. Conventional chest radiography often demonstrates only indirect signs such as recurrent infiltrates, segmental atelectasis, unilateral hyperinflation, or persistent consolidation, while radiolucent foreign bodies may remain undetected [11]. High-resolution computed tomography (HRCT) has significantly improved diagnostic accuracy by enabling precise visualization of endobronchial obstruction, distal air trapping, mucus impaction, post-obstructive consolidation, bronchiectatic changes, and associated parenchymal destruction [11,12]. Consequently, persistent localized pulmonary abnormalities, particularly in patients with a history suggestive of aspiration, should prompt further investigation with computed tomography and diagnostic bronchoscopy.

The composition of the aspirated foreign body considerably influences both the clinical presentation and the subsequent pathological response of the tracheobronchial tree. Organic foreign bodies, including nuts, seeds, and other food particles, usually provoke an intense inflammatory response because of their hygroscopic properties and the release of inflammatory lipids, resulting in early symptom development [3,4]. Conversely, inorganic foreign bodies comprise a heterogeneous group including metallic pins, nails, screws, dental prostheses, orthodontic appliances, pen caps, plastic toys, fragments of medical devices, and various household objects [2,6,13]. Because many inorganic materials are chemically inert, they often produce less pronounced acute symptoms and may therefore remain undetected for prolonged periods. Nevertheless, persistent endobronchial obstruction ultimately results in chronic infection, excessive granulation tissue formation, and progressive structural destruction of the distal airways [7,9].

Plastic foreign bodies present an additional diagnostic challenge because most are radiolucent and cannot be directly identified on conventional chest radiography [11]. Metallic foreign bodies are usually radiopaque and therefore more readily detected; however, prolonged exposure to the humid inflammatory environment of the bronchial tree may result in progressive surface corrosion, oxidation, and partial degradation of their metallic components. These changes increase local tissue irritation, promote granulation tissue formation, and may complicate bronchoscopic extraction because of firm impaction within the bronchial wall [9,14]. Although corrosion of aspirated metallic foreign bodies has occasionally been described, detailed reports documenting these changes remain relatively scarce in the literature.

Among inorganic foreign bodies, aspiration of drawing pins (thumbtacks or push pins) represents an exceptionally rare clinical entity, particularly in adolescents. Most published reports describe sewing needles, scarf pins, dental materials, or orthodontic appliances, whereas chronic retention of a drawing pin has only rarely been documented. The combination of prolonged endobronchial retention, progressive corrosion of the metallic component, extensive granulation tissue formation, and secondary saccular bronchiectatic changes represents an unusual clinical scenario with limited representation in the available literature.

**Aim:** To present and analyze a rare clinical case of a three-year retained endobronchial metallic foreign body (a push pin) in an adolescent, associated with secondary saccular bronchiectatic changes, and, based on this case, to summarize the diagnostic and therapeutic lessons through a structured review of comparable published cases.

## 2. Case Presentation

A 16-year-old male patient was admitted to the clinic following imaging findings (chest X-ray—Figure 1, and CT scan—Figure 2 and Figure 3) indicating the presence of a foreign body in the tracheobronchial tree. The imaging examinations were performed at the request of a pediatrician because of a three-year history of persistent cough that had failed to respond to treatment with antibiotics, bronchodilators, and antitussives.

**Medical History:** The medical history was obtained from the patient and his mother; communication was limited by a language barrier, and additional information was therefore obtained from the accompanying medical documentation. The patient had a three-year history of recurrent bronchitis and pneumonia, which had been treated mainly on an outpatient basis with antibiotics (no exact data on the number of episodes). During this period, he was not hospitalized, and no imaging studies were performed. A persistent cough was also present between episodes of illness, although it was less pronounced during these periods. At another visit to the pediatrician for a persistent cough, a chest X-ray was performed, revealing the presence of a foreign body in the tracheobronchial tree. One day later, a chest CT scan was performed, which confirmed the finding. Following identification of the foreign body, the patient admitted to an episode of choking on a push pin intended for a corkboard that had occurred three years earlier. According to the patient, he had not reported the incident at the time for fear of being punished. The patient was referred for hospitalization for removal of the foreign body.

Clinical findings—The patient was in good general condition and afebrile. The oral, nasal, and pharyngeal mucosae were pink. No enlarged cervical lymph nodes were palpable. The nasal passages were patent, with no pathological nasal discharge. Auscultation of the lungs revealed accentuated vesicular breath sounds without wheezing or other adventitious sounds. Cardiac activity was regular. Laboratory investigations were within normal limits.

Imaging Findings—Computed tomography (CT) of the chest revealed a metallic, needle-shaped foreign body located within the right lower lobe bronchus (Figure 2 and Figure 3). Distal to the foreign body, multiple saccular bronchiectatic changes were identified, with air–fluid levels present in some of them.

After collecting all historical, clinical, laboratory, and imaging data, the patient’s condition was discussed at a multidisciplinary meeting involving a pediatrician, a pediatric surgeon, and an ENT specialist. A decision was made to attempt endoscopic removal of the foreign body, with readiness to proceed to open surgical intervention in case of failure. Postoperative management of the parenchymal changes caused by the foreign body was planned in the Department of Pediatrics. Written informed consent for the procedure was obtained from the patient’s parents.

The removal of the foreign body was performed by a specialized bronchoscopy ENT team. Under general anesthesia, the patient’s tracheobronchial tree was accessed using a rigid bronchoscope. A push pin whose metal tip had a rusty color was found in the right lower lobe bronchus (Figure 4). Granulation tissue partially covering the foreign body was also visualized. Removal was carried out using endoscopic forceps. The removed foreign body is shown in Figure 5. During extraction, part of the metal tip of the foreign body fragmented inside the bronchoscope tube (Figure 6).

Following extraction, there was scant bleeding from the impaction site, which stopped spontaneously within 3 min. Approximately 3 mL of purulent secretion retained distal to the foreign body was aspirated (Figure 7).

After removal of the foreign body, a complete inspection of the tracheobronchial tree was performed, and no other foreign bodies or remnants of the extracted push pin were found. When the extracted push pin was compared with an unused push pin of the same type, a marked reduction in its metal component was observed, whereas the plastic head showed no visible change (Figure 8).

The postoperative period was uneventful: the patient remained afebrile and hemodynamically stable, with vesicular, slightly harsh breath sounds. The cough persisted.

On the recommendation of the pediatricians, postoperative treatment consisted of ceftriaxone 2 g [intravenously] every 12 h, nebulized salbutamol 0.3 mL three times daily, and methylprednisolone 20 mg [intravenously] twice daily. Forty-eight hours after the extraction, the child was transferred for further treatment to the Department of Pediatrics, where conservative treatment was continued unchanged for another 7 days, after which the child was discharged in good general and local condition with home therapy consisting of oral clindamycin 600 mg three times daily for 10 days, followed by oral azithromycin 500 mg every third day for 3 months, and oral erdosteine 300 mg twice daily for 3 months. Long-term macrolide treatment was chosen because of the documented bronchiectatic changes and the three-year history of recurrent lower respiratory tract infections, with the aim of reducing the risk of further exacerbations; the mucolytic erdosteine was added to facilitate airway clearance of the retained secretions [15].

Follow-up. The patient did not attend the scheduled follow-up visit at three months after removal of the foreign body, for which a follow-up CT scan and microbiological examination of a throat swab had been planned; therefore, the persistence or reversibility of the complaints, and especially of the bronchiectatic changes, could not be assessed.

The exact timeline of the case is shown in Table 1.

## 3. Discussion

Foreign body aspiration into the tracheobronchial tree is a well-known clinical problem in childhood, but the presented case has a number of distinctive features that set it apart from the typical clinical picture. Most aspiration episodes occur in young children, particularly those under 3 years of age, whereas in older children and adolescents this condition is significantly rarer [1,3,4]. The present case concerns a 16-year-old patient with a metallic foreign body retained in the right lower lobe bronchus for three years. The duration of retention and the lack of timely recognition of the aspiration episode are the main features determining the subsequent pathological changes.

The lack of diagnostic recognition in this case led to the development of bronchitis and pneumonia, treated mainly on an outpatient basis with antibiotics, with the cough persisting between the individual episodes as well. This disease course is characteristic of cases involving missed or delayed diagnosis of a foreign body, in which the initial aspiration episode may be brief and go underestimated, while the subsequent nonspecific respiratory symptoms are interpreted as recurrent infections or other more common pulmonary diseases [2,4,5,6]. It has been described in the literature that diagnostic delay can range from several months to years and may be associated with the development of irreversible pulmonary damage [2,8]. The persistent presence of a foreign body disrupts mucociliary clearance and leads to secretion retention, recurrent bacterial infections, and a chronic inflammatory process. Over time, granulation tissue forms around the foreign body, and prolonged inflammation and infection can lead to destruction of the elastic and muscular structures of the bronchial wall and to irreversible bronchial dilation [7,9]. It is likely precisely this mechanism that underlies the multiple saccular bronchiectasis found distal to the site of obstruction in our patient, some of which contained fluid.

Another important aspect of this case is the nature of the foreign body. Unlike organic foreign bodies, which often provoke a more pronounced early inflammatory reaction, inorganic materials can be relatively inert and cause more limited initial symptoms [3,4,6]. This may contribute to their prolonged non-recognition. In the present case, the foreign body is a metal push pin, fixed in the lower lobe bronchus. Bronchoscopy revealed partial coverage by granulation tissue, which is consistent with the chronic local reactions around long-retained foreign bodies described in the literature [7,9].

Of particular interest is the change observed in the metal part of the push pin. After a three-year stay in the tracheobronchial tree, the metal tip had a rusty color, and part of it fragmented during extraction. Comparison with an identical, unused push pin showed a marked reduction in its metal portion, while the plastic component showed practically no change. This finding suggests marked corrosive and structural degradation of the metal under conditions of prolonged retention in a moist and inflammatorily altered bronchial environment. Corrosion and oxidation can increase local irritation and granulation tissue formation, while also complicating endoscopic extraction [9,14]. Nevertheless, detailed descriptions of significant metal degradation in long-retained endobronchial foreign bodies remain relatively limited in the available literature, which adds further interest to the present case.

Imaging diagnostics play an essential role in establishing the diagnosis and assessing secondary pulmonary changes. Conventional radiography can show the presence of a radiopaque foreign body or indirect signs of bronchial obstruction, including infiltrative changes, atelectasis, unilateral hyperinflation, or persistent consolidation, but its ability to evaluate distal structural changes is limited [11,16]. In the present case, the metallic foreign body was visualized on radiography, and the subsequent computed tomography allowed precise localization in the right lower lobe bronchus, along with the simultaneous identification of multiple distal saccular bronchiectases with fluid. This example demonstrates the importance of CT examination in patients with persistent or recurrent pulmonary changes localized to the same site and an insufficient response to standard treatment [16].

The choice of therapeutic approach in such cases is determined by the location and nature of the foreign body, its prolonged retention, and the presence of secondary pulmonary changes. Endoscopic removal under general anesthesia with a rigid bronchoscope allows direct visualization of the foreign body, assessment of the local reaction, and removal of retained secretions. Rigid bronchoscopy remains an important method for extracting tracheobronchial foreign bodies, particularly in children, owing to the possibility of airway control and the use of a variety of extraction instruments [1,3,7].

**Comparison with published cases.** Published data on foreign body aspiration in children show a predominance of organic over inorganic foreign bodies [3]. Information on inorganic bronchial foreign bodies retained for more than two years in children consists mainly of single case reports and small case series [17,18,19,20,21,22,23]. Table 2 presents selected pediatric cases of long-retained inorganic airway foreign bodies published in recent years, together with a pediatric series of push pin (thumbtack) aspiration, identified through a targeted search of PubMed and the Directory of Open Access Journals (DOAJ). Most long-retained inorganic foreign bodies in children were plastic or composite objects, such as pen caps, whistles, or the metal end-piece of a pencil. Retention for 4–7 years was associated with atelectasis, bronchiectasis, and bronchial stenosis [19,20,21]. Localized bronchiectasis distal to retained foreign bodies has also been described in a pediatric series [19,23]. In the pediatric thumbtack series, the main difficulty was extraction, because the flat head and sharp point predispose to entrapment in the bronchial mucosa; rigid bronchoscopy combined with balloon dilation allowed removal without complications [20]. In this limited search, we did not identify a published pediatric case of a push pin retained for several years with corrosion and fragmentation of its metallic component. The study by Thornton and Yunker (2015) is the only one in Table 2 that also describes push pin aspiration; however, the retention time was not reported, at least one case presented acutely, and no chronic pulmonary complications were described [20].

The persistent cough after extraction demonstrates that removing the cause of obstruction does not immediately resolve all clinical manifestations. In this patient, bronchiectatic changes resulting from prolonged obstruction and recurrent infection were already present at diagnosis. Postoperative antibiotic and symptomatic therapy were therefore required, followed by continued prophylaxis after discharge, in line with current recommendations for children and adolescents with bronchiectasis [15]. Whether these changes will persist or partially regress after relief of the obstruction can only be established through longer-term clinical and radiological follow-up.

## 4. Conclusions

The present case represents a rare combination of three-year retention of a metallic endobronchial foreign body in an adolescent, marked corrosive degradation of the metal component, development of granulation tissue, and secondary saccular bronchiectatic changes. The case underscores the need for a high degree of clinical suspicion in patients with chronic cough and recurrent pulmonary infections, particularly when symptoms are persistent or localized to the same site. Timely imaging diagnostics and bronchoscopic evaluation may help limit the progression of obstructive and infectious-inflammatory changes and the risk of potentially irreversible pulmonary damage.

## Figures and Tables

**Figure 1:**
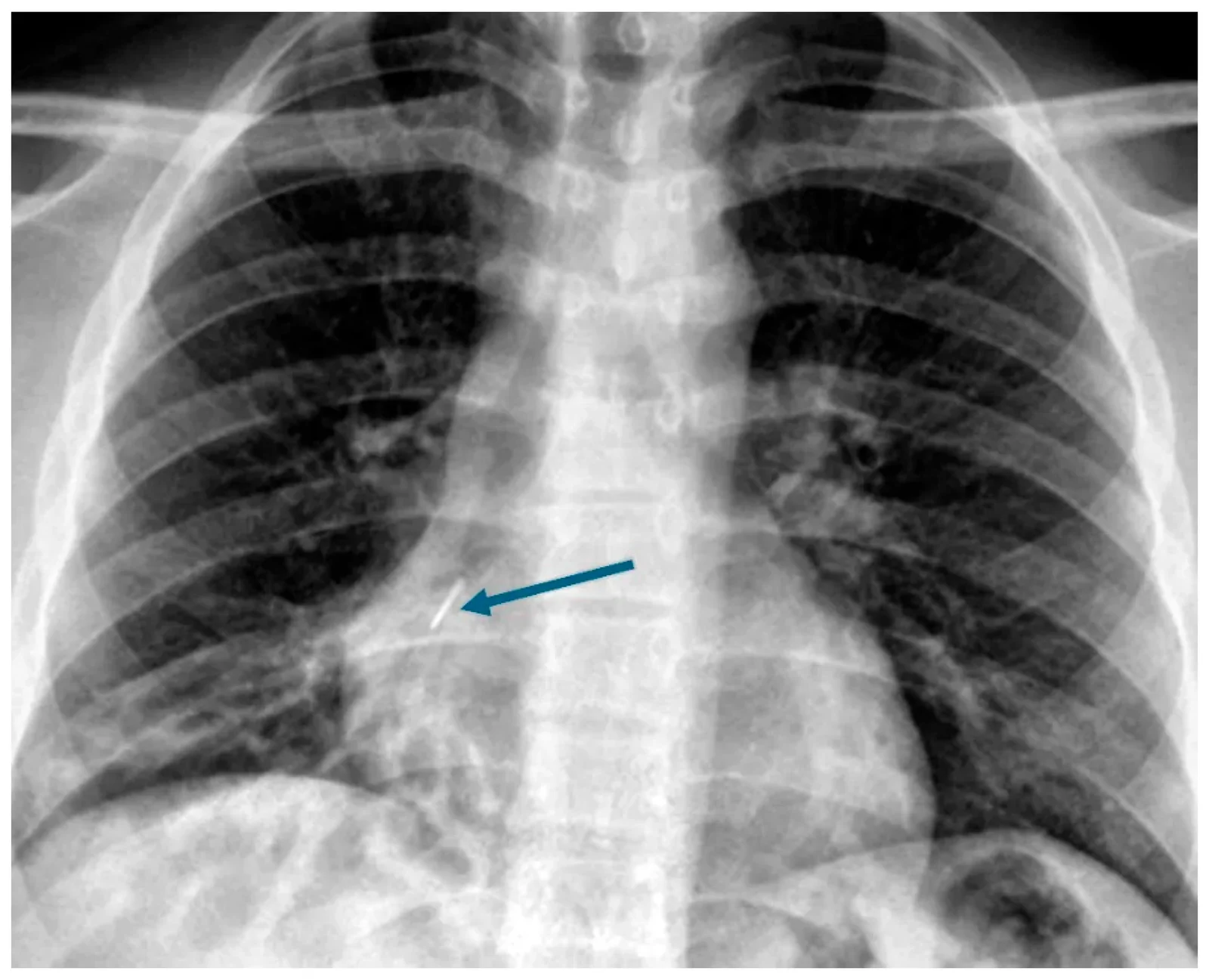
Chest radiograph showing the radiopaque foreign body (arrow).

**Figure 2:**
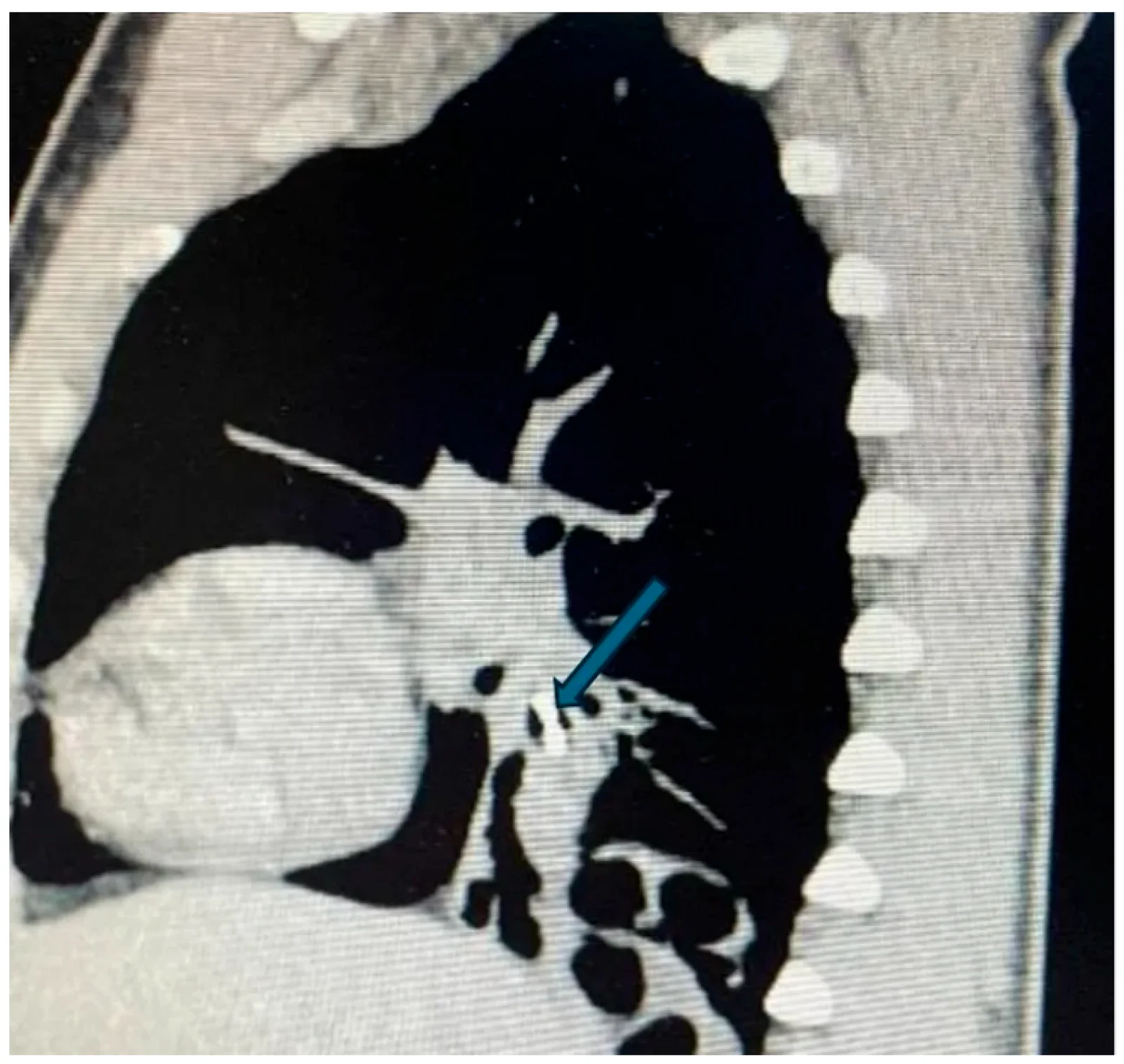
Chest CT, parasagittal reconstruction, showing a metallic foreign body (arrow) in the right lower lobe bronchus.

**Figure 3:**
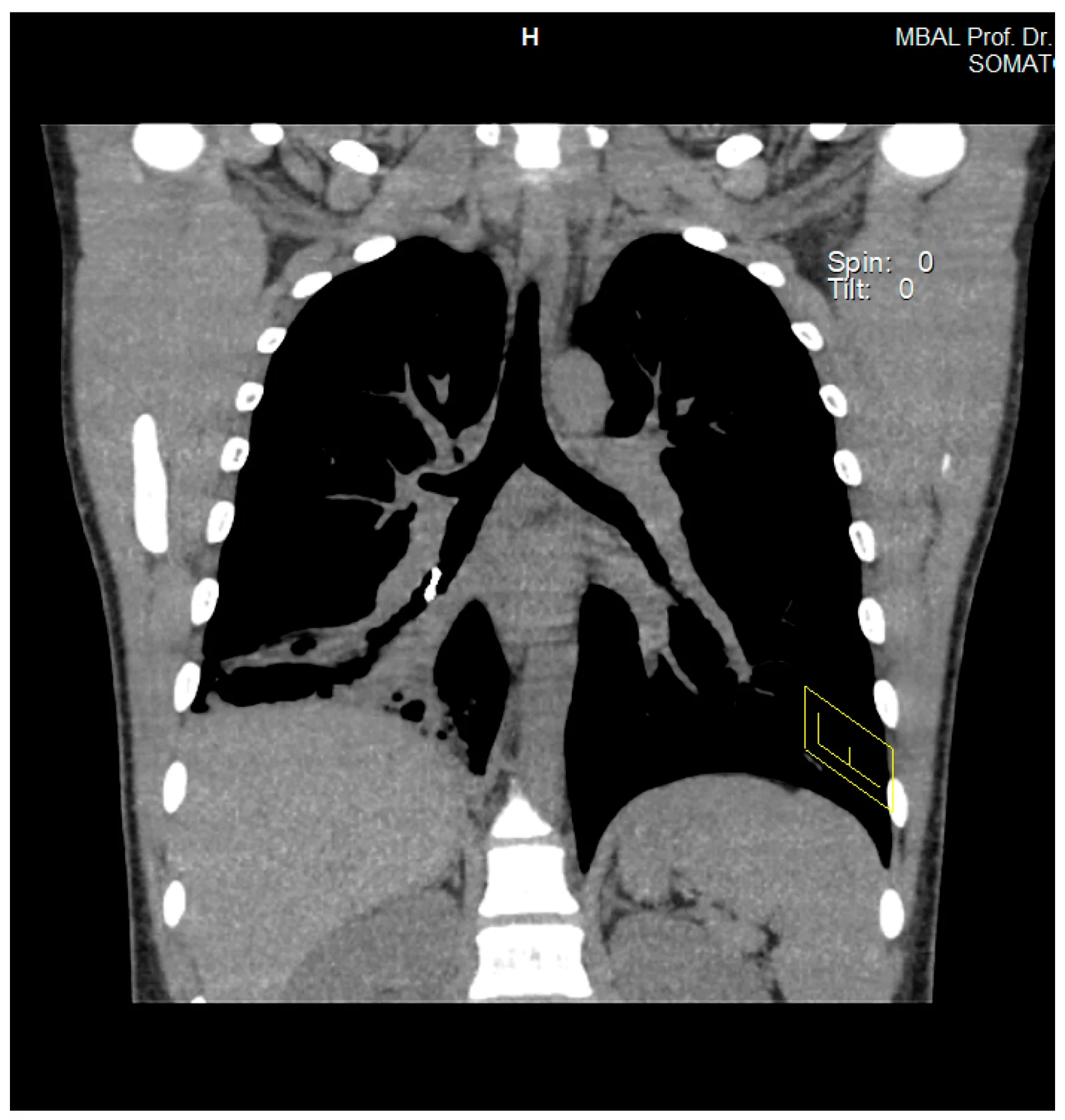
Chest CT, coronal reconstruction, showing a metallic foreign body in the right lower lobe bronchus.

**Figure 4:**
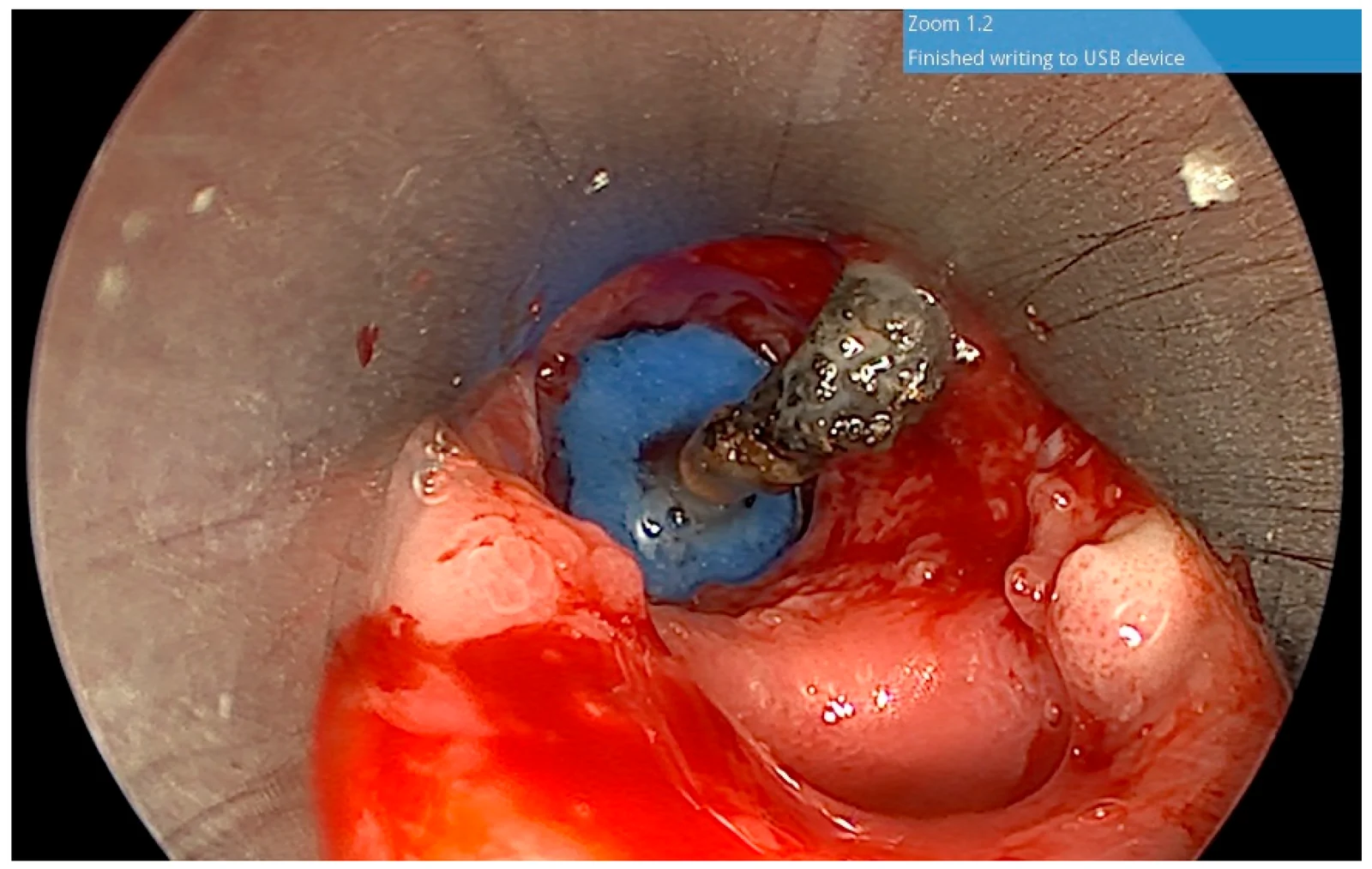
Bronchoscopic view of the foreign body in the right lower lobe bronchus.

**Figure 5:**
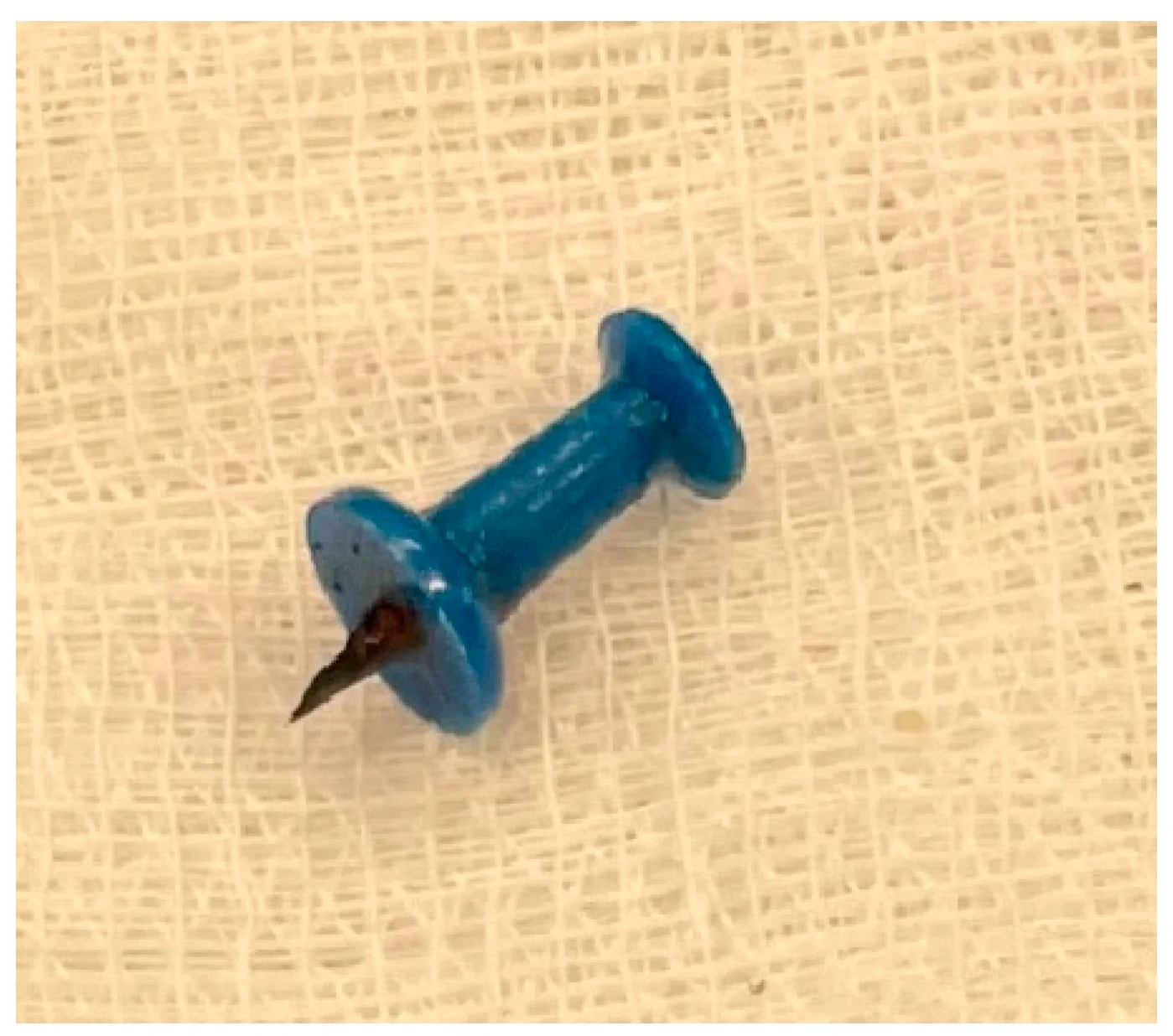
The extracted foreign body.

**Figure 6:**
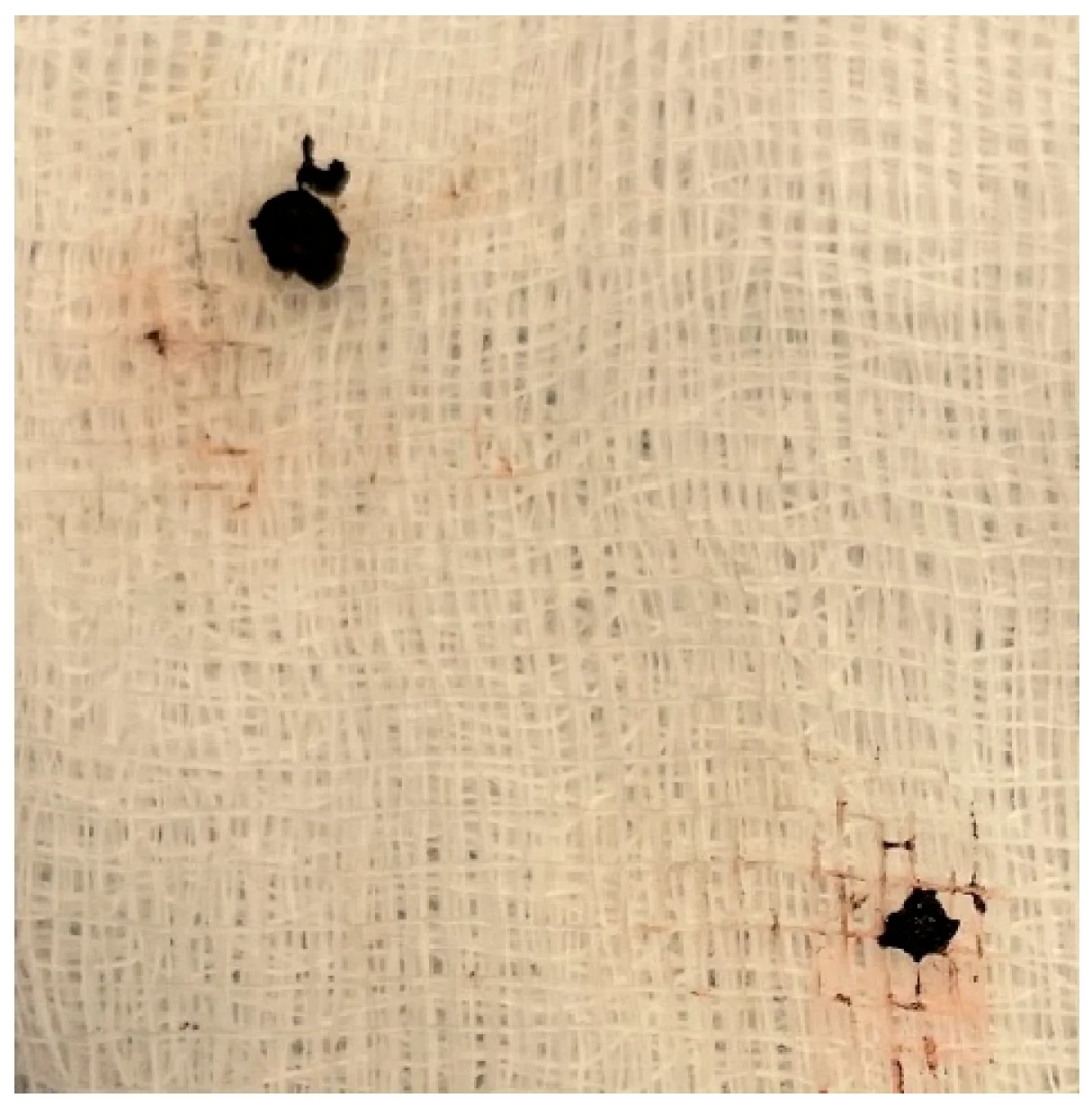
Metal fragments that detached during extraction.

**Figure 7:**
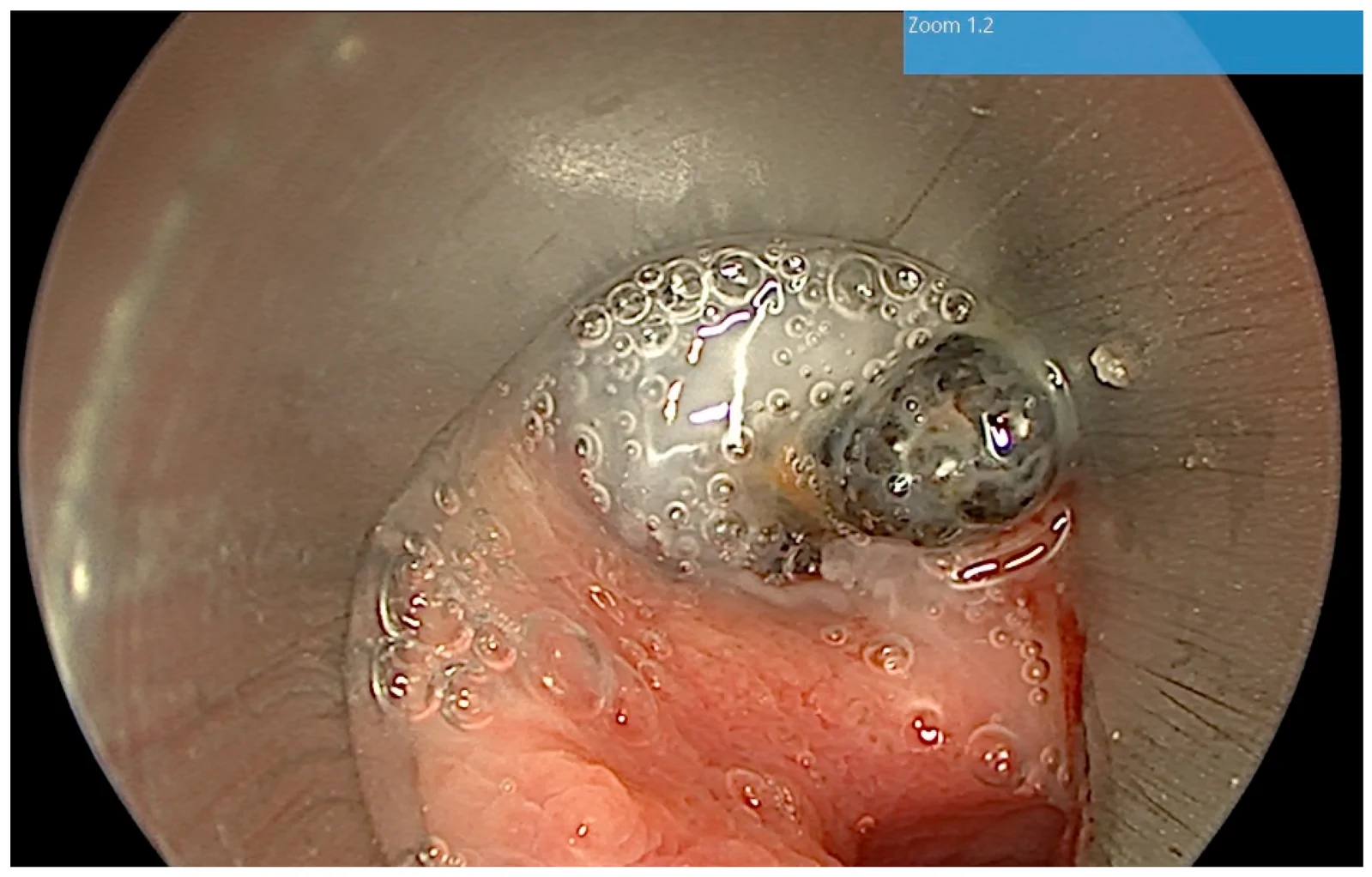
Purulent secretions in the bronchi distal to the foreign body.

**Figure 8:**
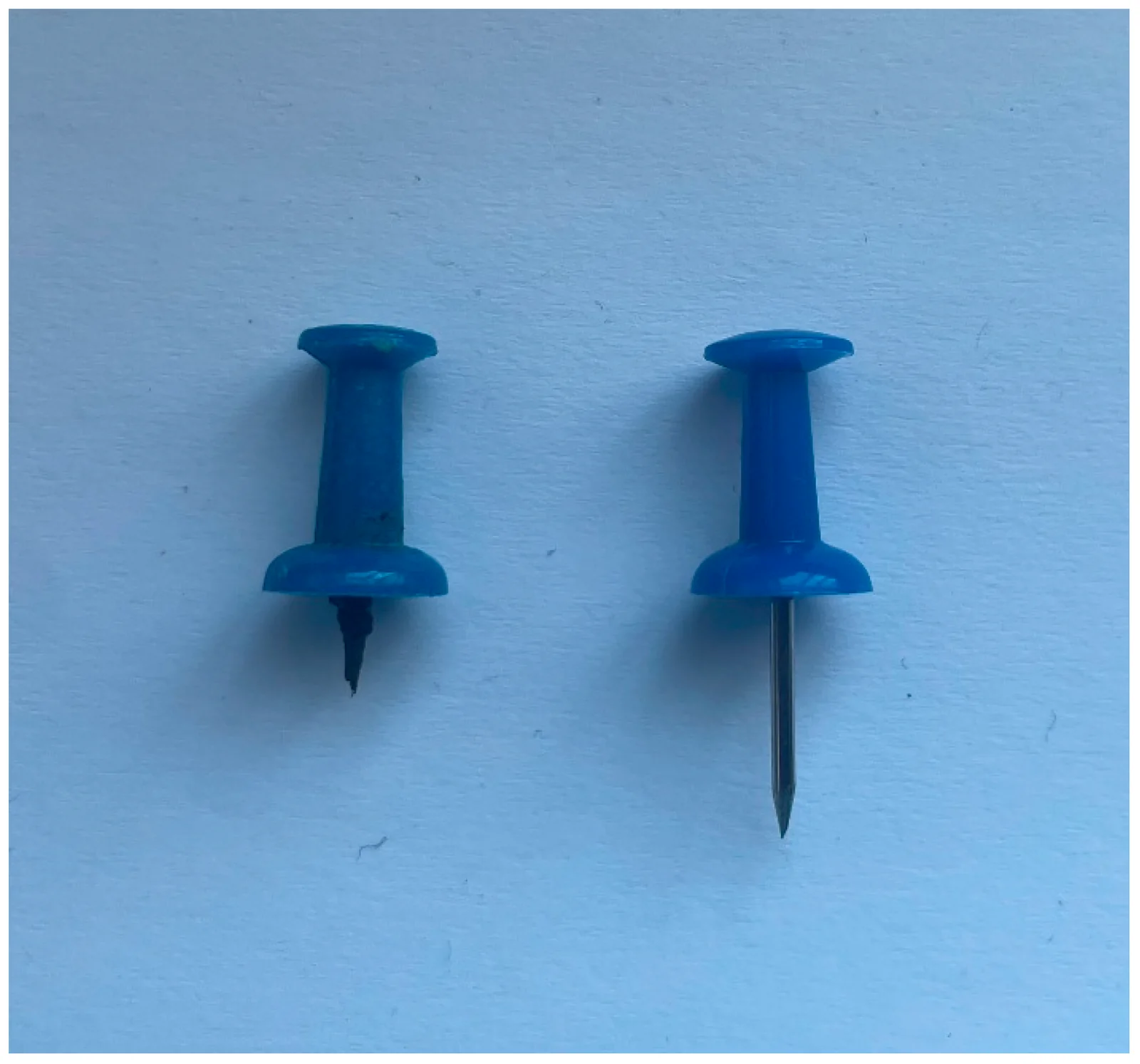
Comparison of the extracted push pin with an unused push pin of the same type.

**Table 1:** Timeline of the case.

Time Point	Event
≈3 years before admission	Choking episode on a corkboard push pin; not reported at the time.
Following 3 years	Recurrent bronchitis and pneumonia, treated mainly with outpatient antibiotics, with persistent cough between episodes; no exact data on the number of episodes are available.
[22 June 2026]	Chest radiograph requested by pediatrician for persistent cough unresponsive to treatment: radiopaque foreign body.
[23 June 2026]	Chest CT: needle-shaped metallic foreign body in the right lower lobe bronchus; distal saccular bronchiectatic changes with air–fluid levels.
Day 0	Hospitalization; multidisciplinary evaluation; rigid bronchoscopy and extraction under general anesthesia. Conservative treatment initiated.
Day 2	Transfer to the Department of Pediatrics.
Day 9	Discharge; oral clindamycin for 10 days, then azithromycin and erdosteine for 3 months.
Follow-up	The patient did not attend the scheduled follow-up

**Table 2:** Published pediatric cases of long-retained inorganic airway foreign bodies and push pin aspiration, compared with the present case.

Reference	Age, Sex	Foreign Body	Duration	Location	Pulmonary Complications	Extraction	Outcome
Lumaya and Mohammed, 2016 [17]	11 y, M	Metal end-piece of a pencil with eraser	6 years	Distal left main bronchus	LLL atelectasis, LUL consolidation; initially treated as tuberculosis	Rigid bronchoscopy, 2 sessions (first stopped for bleeding from granulation tissue)	Marked clinical and radiological improvement at discharge
Razafimanjato et al., 2021 [18]	11 y, F	Plastic pen cap	4 years	Left main bronchus	Bronchiectasis, atelectasis, recurrent pneumonia, hemoptysis; destroyed left lung	Left pneumonectomy (bronchoscopic extraction not available)	Asymptomatic 2 years after surgery
Nongpiur et al., 2020 [19]	17 y, M	Plastic whistle	7 years	Right intermediate bronchus	RML and RLL collapse, bronchiectasis, web-like stenosis; hemoptysis	Rigid bronchoscopy with electrocautery incisions	Collapse resolved at 1 month; mild residual stenosis
Thornton and Yunker, 2015 [20]	3 children: 2 y 7 mo, 9 y, 15 y (all M)	Thumbtacks (push pins)	The exact duration was not specified in the article.	Right main bronchus, left main bronchus, LLL bronchus	Hypoxic arrest at aspiration in 1 case;	Rigid bronchoscopy; failed forceps and topical epinephrine, then balloon dilation (5 Fr atrioseptostomy catheter) and forceps	Removed in all, no iatrogenic complications; 1 death from prior hypoxic injury
Hakim et al., 2020 [21]	6 y, M	Metal screw	4 years (by history of swallowing)	Superior branch of left bronchus	Pneumonia, partial LUL atelectasis, abscess in posterior basal segment of LLL	Bronchoscopy; residual brittle metal debris and pus removed by thoracotomy	Improved; discharged on day 15 after PICU care
Mishra et al., 2022 [22]	12 y, M	LED bulb (plastic cap with metal filaments)	3 years (symptom duration; aspiration not recalled)	Left main bronchus, ~2 cm from carina	Recurrent chest infections; partial left volume loss, right hyperinflation; complete occlusion with granulation tissue	Flexible bronchoscopy via LMA under GA, biopsy forceps, en bloc removal	Symptom-free at 4 weeks
Noujeim, 2023 [23]	12 y, M	Plastic pen tip	~2 years (from first symptoms; no choking history)	Left main bronchus	Recurrent infections; lingular/LLL consolidation, abscesses, bronchiectatic changes; granulation tissue	Flexible bronchoscopy under sedation, Fogarty balloon + forceps	Marked lung re-expansion at 6 months; residual localized bronchiectasis
**Present case**	**16 y, M**	**Metal push pin with corroded tip**	**3 years**	**RLL bronchus**	**Multiple saccular bronchiectatic changes with air–fluid levels; granulation tissue**	**Rigid bronchoscopy with forceps; metal tip fragmented**	**Persistent cough at discharge; no follow-up**

**Abbreviations:** y, years; mo, months; M, male; F, female; LUL, left upper lobe; LLL, left lower lobe; RML, right middle lobe; RLL, right lower lobe; LMA, laryngeal mask airway; GA, general anesthesia; PICU, pediatric intensive care unit; LED, light-emitting diode.

## Data Availability

The data presented in this study are available on request from the corresponding author. The data are not publicly available due to restrictions, e.g., privacy or ethical.
